# Clinico‐Epidemiological and Molecular Evidences for Reactivation of Herpesviruses in Dapsone‐Induced Hypersensitivity Reactions in Nepalese Leprosy Patients: An Observational Study

**DOI:** 10.1002/iid3.70385

**Published:** 2026-02-25

**Authors:** Divya RSJB Rana, Jivan Shakya, Suwash Baral, Reejana Shrestha, Kishor Koju, Jarina Joshi, Deanna A. Hagge, Mahesh Shah

**Affiliations:** ^1^ Mycobacterial Research Laboratory, Anandaban Hospital The Leprosy Mission Nepal Lalitpur Bagmati Province Nepal; ^2^ Central Department of Biotechnology Tribhuvan University Kirtipur Nepal; ^3^ Anandaban Hospital The Leprosy Mission Nepal Lalitpur Bagmati Province Nepal

## Abstract

**Introduction:**

Dapsone hypersensitivity (DHS) is a potentially fatal and severe cutaneous adverse reaction that occurs in patients taking dapsone. As leprosy, for which dapsone is used as part of multidrug therapy, usually occurs in countries with resource limitations, the morbidity and mortality caused by DHS are more dreadful. Herpesviruses (especially HHV‐5 and 6) are frequently associated with the etiology of drug‐induced hypersensitivity reactions, and the reactivation of these viruses coincides with the reflare of clinical symptoms even after the cessation of culprit drugs.

**Methods:**

We reviewed the hospital charts of patients (Cohort 1) with DHS who were admitted to our hospital at the time of the DHS episode. Similarly, we examined the presence of HHV‐5 and 6 in another independent group of DHS blood samples (Cohort 2) by PCR.

**Results:**

Seventy‐one percent (71%, 17/24) of DHS patients experienced recrudescence (reflare) of symptoms ~20 days after cessation of dapsone in Cohort 1. In Cohort 2, 39% (13/33) of blood samples from DHS patients showed the presence of at least one herpesvirus.

**Conclusion:**

In this exploratory study, our data suggest the role of herpesviruses in the natural history of DHS and identify patterns that support future hypothesis‐driven investigations into antiviral treatment strategies for the management of DHS cases, with the potential to reduce morbidity and mortality.

## Graphical abstract

This study found clinical and molecular evidences for the presence of herpesvirus (HHV‐5 and HHV‐6) reactivation during dapsone‐induced hypersensitivity in Nepalese leprosy patients. Reflare of hypersensitivity symptoms occurred on average 20 days after culprit drug cessation. Patients with reflare (71%) had more abnormal laboratory values (but not statistically significant).

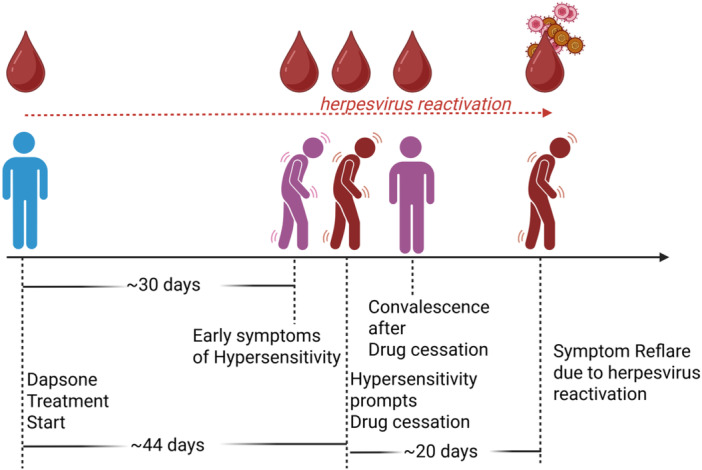

## 1 Introduction

Leprosy is an infectious disease caused by *Mycobacterium leprae* and/or *Mycobacterium lepromatosis*. Leprosy bacteria can infect the skin and peripheral nerves, which can lead to stigmatizing sensory and motor impairment, disability, and disfigurement [1]. Dapsone, a sulfone drug, was the first antibiotic developed to treat leprosy (1940s); and, since the early 1980s, dapsone has remained one of the three drugs (with rifampicin and clofazimine) in the World Health Organization (WHO) recommended multidrug therapy (MDT) to treat leprosy worldwide. Currently, Dapsone is approved by the United States Federal Drug Administration for use in only two diseases: leprosy and dermatitis herpetiformis. Nevertheless, it is also used in a wide variety of other conditions, including malaria prophylaxis, autoimmune dermatological diseases, *Pneumocystis jiroveci* pneumonia in AIDS patients, and brown recluse spider bites [2].

Dapsone‐induced hypersensitivity was first described in 1949 [3]; and since then, DHS has been variably called dapsone syndrome, sulfone syndrome, 6 week syndrome, and dapsone hypersensitivity (DHS, which will be used herein). Drug‐induced hypersensitivities, such as DHS, are also described as Drug Reaction with Eosinophilia and Systemic Symptoms (DRESS) that are typical across different drugs, including allopurinol, abacavir, carbamazepine, and sulfamethoxazole [4]. Symptoms may include fever, whole body skin rash, and enlarged lymph nodes [4, 5, 6, 7].

As the majority of global dapsone usage is for leprosy, the development of DHS criteria and related investigations has predominantly focused on leprosy cases. The current diagnostic criteria for DHS were established by Richardus and Smith [6]. The worldwide prevalence of DHS is estimated to be ~1.4% with a mortality rate of 10% [5]. As with most drug‐induced hypersensitivities, DHS treatment consists of cessation of the culprit drug (dapsone), high‐dose corticosteroids treatment then tapered individually as needed, and supportive treatment for other symptoms as per individual needs [5, 8]. The first few weeks of DHS treatment are critical, during which the severity of the syndrome may escalate. Recovery often involves months of specialized inpatient care, followed by potentially more months at home. In 2013, two groups in China independently reported the association of DHS with a human leukocyte antigen HLA‐B*13:01 [9, 10]. Though the association has been validated in multiple ethnicities/countries like Taiwan, Thailand, South Korea, India, and Indonesia [11], the factors affecting the severity of DHS have rarely been studied. Two in silico [12, 13] and one in vitro study [14] postulated the requirement for binding of dapsone to F pocket of peptide binding groove of HLA‐B*13:01 in the stimulation of T cells during DHS.

In the 1990s, an association was reported between drug‐induced hypersensitivity, or DRESS in general, and reactivation of human herpesvirus 6 (HHV‐6) [15, 16]. Since then, there has been repeated and confirmatory data on the presence/reactivation of HHV‐6 and other related viruses [17, 18, 19, 20]. Clinically, these reactivations manifest as a “reflare” of symptoms, typically within the first few weeks after cessation of the culprit drugs [21, 22]. During a “reflare,” symptoms such as exfoliation and jaundice reappear for no obvious reasons. Ishida's group studied the DNA viral loads for Epstein–Barr virus (EBV), Cytomegalovirus (CMV), and HHV‐6 in patients of DiHS/DRESS, SJS, TEN, and SJS/TEN [23]. HHV‐6 viral loads peaked ranging from the 11th to 100th day for DiHS/DRESS, and at least 50% of the studied patients developed reactivation. Based on data that only up to 60% of DRESS patients had HHV‐6 reactivation, Roujeau argued that HHV‐6 reactivation was not an invariable or specific criterion for DRESS [17]. However, Shiohara et al. [20] asserted that viral detection kits are less available in European countries, and inaccurate timing for assessments of viral reactivation could be the cause for the lack of association between HHV‐6 reactivation and DiHS/DRESS in European studies [20]. The Japanese Consensus Group uses the presence of HHV‐6 as the defining criterion for drug‐induced hypersensitivities. In case the HHV‐6 is absent due to inappropriate timing or any other reasons, the DiHS is considered “atypical” [19, 20]. In leprosy, DHS has rarely been reported alongside HHV‐6 or other related viruses. A study from Japan reported 100 drug hypersensitivity cases, among which 5 had DHS, and 60% (3/5) were reported to have developed HHV‐6 reactivation (Toyhama 2007). A study in Taiwan assessed HHV‐6 and EBV viral loads by real‐time PCR during the acute phases of six dapsone hypersensitive patients, but none indicated viral presence [24]. Herpesvirus reactivation has been reported in DHS in case reports [25, 26].

Shiohara et al. [20] have reviewed the reactivation phenomenon in drug allergies across different drugs and hypothesized that plasma IgG concentration is very low during the acute phase of drug allergies (3–11 days of onset) and this may reactivate HHV‐6 [20]. Thus, the concentration of HHV‐6 may only increase during the late acute and early subacute phases (11–36 days of allergy onset) [20, 23]. As Chen et al. [24] sampled patients only at DHS onset, this could be the reason why they were unable to detect HHV‐6 in the DHS patients. In addition to HHV‐6, other drug‐induced hypersensitivity research has indicated that human herpesvirus 5 (HHV‐5, Cytomegolovirus or CMV) is associated with increased severity of drug‐induced hypersensitivity symptoms [26]. A Nigerian study as early as 1951 [27, 28] also found that DHS was usually seen in leprosy patients who had glandular fever or infectious mononucleosis. Infectious mononucleosis is caused by EBV/HHV‐4, a close relative of HHV‐6, and has been seen in DiHS/DRESS patients [23].

In this study, we retrospectively analyzed patient data from medical charts to determine whether a “reflare” of DHS symptoms was noted in the first few weeks after cessation of dapsone. Additionally, an independent group of available patient blood samples collected after DHS onset was examined for the presence of HHV‐5 and HHV‐6 by qPCR.

## 2 Material and Methods

**Figure 1:**
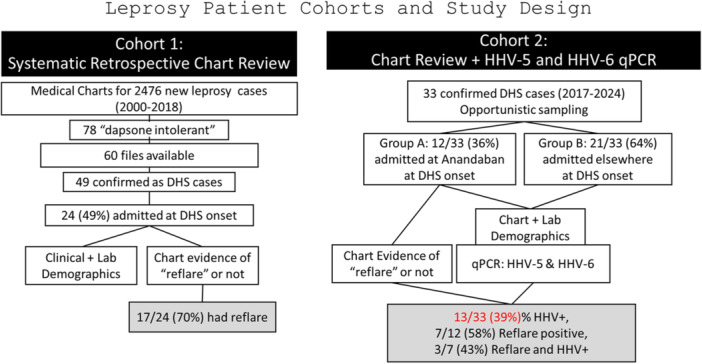
Study design showing patient enrollments in two cohorts studied.

This study consisted of two independent cross‐sectional studies (Cohort 1 and Cohort 2) conducted in the same hospital setting (see Figure 1). The first one (Cohort 1) was a cross‐sectional study of clinical features of DHS cases based on a retrospective medical charts review. This is intended to clarify the clinical epidemiology (explained below) related to DHS due to herpesvirus reactivation. The second cross‐sectional study (Cohort 2) examined the presence of herpesviruses in a set of blood samples collected from DHS patients. This study provided molecular evidence for the presence of the herpesviruses in DHS patients. This study is reported according to the STROBE Statement—checklist of items that should be included in reports of observational studies [29].

### 2.1 Patient Populations and Definitions for Retrospective Chart Review

All participants in the study were patients of Anandaban Hospital, Lalitpur, Nepal. The sample sizes were based on all available medical charts from 2000 to 2018 (Cohort 1) and opportunistic blood samples taken between 2017 and 2024 (Cohort 2). As the hospital is a tertiary‐level referral center for leprosy complications in Nepal, DHS cases included (Cohort 2) both leprosy cases diagnosed and treated at Anandaban Hospital and those diagnosed and treated elsewhere but referred to Anandaban for complication management (e.g., DHS). All clinical and demographic details of the patients at Anandaban Hospital are recorded in the medical charts. All patients in this study were diagnosed according to WHO guidelines and treated with MDT consisting of monthly rifampicin (600 mg), daily dapsone (100 mg), and daily clofazimine (monthly 300 mg, daily 50 mg) [30]. The recommended treatment duration is 1–2 years for multibacillary leprosy and 6 months for paucibacillary leprosy. The occurrence of leprosy reactions was confirmed using WHO Guidelines [31] and reaction episodes were excluded from reflare analyses.

DHS was confirmed using the Richardus and Smith criteria [6], which included the following four features: (i) initiation of symptoms from the 2nd to 8th week after the start of dapsone; (ii) presence of at least two of the four symptoms: skin rashes, jaundice, lymph node swelling, and fever; (iii) no other drug allergy/hypersensitivity or other diseases; and (iv) no leprosy reactions. In cases where DHS patients were referred from other clinics, the diagnosis of DHS was confirmed based on available patient documents and/or patient experiences. DHS was always confirmed by at least one or more experienced leprologist dermatologists working in the leprosy field/hospital for ≥ 5 years. All DHS cases were treated as per the recognized standards of care [32] for DRESS, which includes cessation of dapsone, initial high‐dose corticosteroids to be tapered over time as per individual case response, and supportive care as needed for other clinical symptoms. As herpesvirus reactivation as part of DHS pathology was not recognized at our center before this study, no efforts were made to use antivirals for DHS treatment.

### 2.2 Clinical Epidemiology (Cohort 1)

Clinical data related to hypersensitivity episodes were retrospectively collected for DHS patients registered between 2000 and 2018. The data included the timing of MDT initiation, DHS onset, symptoms, and laboratory and symptom profile during hospitalization. Herein, DHS onset is considered the day in which the first DHS signs began, and DHS diagnosis or MDT stop as the day on which DHS was confirmed and dapsone treatment ceased. As reflare of DHS symptoms due to herpesvirus reactivation is known to occur 2–3 weeks after DHS onset [19], special attention was given to chart records to determine whether any reflare or increase in severity of DHS symptoms occurred in the patients during their stay in the hospital. Only patients who had been admitted to Anandaban Hospital during DHS symptoms and those with sufficient clinical data were included in this study. Laboratory parameters related to drug hypersensitivity during the early DHS onset days were also recorded. Further analyses were performed to compare the clinical and laboratory features of patients with or without reflare symptoms.

### 2.3 Molecular Epidemiology of Herpesviruses (Cohort 2)

HHV reactivation can be confirmed by qPCR detection for up to 12 months postreactivation [23]. Therefore, 1–2 mL of patient blood was aseptically collected in EDTA vials opportunistically once per patient after DHS onset from Week 1 up to 12 months later. DNA was extracted from 100 µL of blood using a DNeasy Blood and Tissue Kit (Qiagen, Catalog: 69506). Primers for HHV‐5 [33] and HHV‐6 [34], along with β‐actin [35], were retrieved from the literature. PCR amplification was performed using the Quantitect SYBR Green PCR kit (Qiagen, Catalog: 204143). The primer sequences and reaction conditions are listed in Table 1. Each reaction consisted of 1x Mastermix, 0.2 µM primers, 4 µL template DNA, and water. The specificity of the HHV primers was examined using sequences downloaded from the NCBI Virus database (www.ncbi.nlm.nih.gov/labs/virus/vssi/#/). The qPCR reaction was followed by melting curve analysis to determine the accuracy of the PCR results.

**Table 1:** Sequences of primers used for the detection of herpesviruses (HCMV/HHV‐5 and HHV‐6).

Target	Sequence	Gene	Product size	Reaction condition
HCMV‐F	5′‐GCCCAGGTAGGCCGTTAC‐3′	DNA polymerase catalytic subunit	143	95C: 15 min 40 cycles of: 95C: 15 s 58C: 30 s 72C: 30 s & 72C: 5 min
HCMV‐R	5′‐ATCTGCTGTCCGTCAAAGAT‐3′			
HHV6‐F	5′‐TCGACTCTCACCCTACTGAACGAG‐3′	Large tegument protein deneddylase	163	
HHV6‐R	5′‐TGACTAGAGAGCGACAAATTGGAG‐3′			
ACTB‐F	5′‐GCCCAGGTAGGCCGTTAC‐3′	β‐actin	66	
ACTB‐R	5′‐ATCTGCTGTCCGTCAAAGAT‐3′			

### 2.4 Ethics Approval

The study was conducted according to the prevailing principles of Good Clinical Practice and the Declaration of Helsinki. Formal consent (verbal and written) was obtained prior to sample collection. This study included two cohorts of patients. The first set of patients was part of a retrospective chart review (Nepal Health Research Council, NHRC, Numbered Ref 901/2012). The second cohort of this study, additionally, had their blood samples examined (NHRC Numbered 2391/2022).

### 2.5 Statistical Tests

Different variables between group of patients who showed reflare and those who did not were tested with Mann–Whitney *U* test for nonparametric, unpaired comparisons using R statistical package (RStudio 2023.12.0). As the “wilcox.test” function could not give results due to tied values, “wilcox.exact” was used by installing “exactRankTests” package. Normal distribution of the values was tested using the “shapiro.test” function for the Shapiro–Wilk test. Fisher's exact test was used to compare the proportion of herpesviruses in DHS and dapsone‐naive new leprosy cases.

## 3 Results

### 3.1 Clinical Epidemiology

Between 2000 and 2018, 2476 new leprosy cases were registered at Anandaban Hospital. Cohort 1 was comprised of the 78 cases (78/2476, 3%) with recorded signs of dapsone‐intolerance in that duration, of which 60 were available for review. The types of dapsone intolerance were reevaluated by the first author and, whenever required, by the study clinicians. Forty‐nine (49/60, 82%) were confirmed as DHS cases. Allergy or hypersensitivity status could not be determined for five patients (5/60, 8%); and one patient (1.3%) each had the following conditions: dapsone‐induced photodermatitis, dapsone‐induced agranulocytosis, dapsone‐induced hepatitis, dapsone‐induced methemoglobinemia, dapsone‐induced COPD (with anemia and methemoglobinemia), and dapsone‐induced pneumonia. Finally, we selected the files of 24 patients who were confirmed to have DHS between 2000 and 2018 and were admitted to Anandaban Hospital (The Leprosy Mission Nepal, Lalitpur, Nepal) at the time of DHS onset. Only those who were admitted to the hospital during their full DHS episode were enrolled, as we could analyze all data during the natural history of DHS syndrome and first few months when a reflare associated with HHV could be expected (Table 2).

**Table 2:** Clinico‐demographic characteristics of patients enrolled in the study (Cohort 1).

Variables		Males	Females	Total
Number		15	9	24
Average age (years)		35.4	41.5	37.7
Age Range (years)		15–77	15–67	15–77
Comprehensive Ridley Jopling class	TT	1	0	1
	BT	3	4	7
	BL	3	1	4
	LL	5	2	7
	PN	1	1	2
	NA	1	1	2
	Not leprosy*	1	0	1
% Neg BI		43% (6/14)	44% (4/9)	43% (10/23)
% PB treatment		27% (4/15)	22% (2/9)	25% (6/24)
Earlier allergy history		13% (2/15)	11% (1/9)	13% (3/24)
Average onset days		24.9	37.3	29.8
Onset range (days)		2–37	2–130	2–130
MDT start to stop (days)		41.9	48.7	44.4
MDT start to stop, range (days)		4–103	3–145	3–145
Reaction steroid tmt before DHS		40% (6/15)	22% (2/9)	33% (8/24)
DHS onset to admission (days)		15.5	17.9	16.4
*Reflare data*				
Reflare seen		73% (11/15)	67% (6/9)	71% (17/24)
MDT stop to reflare (days)		17.4	24.8	20
MDT stop to reflare, range (days)		5–56	4–45	4–56
DHS onset to reflare (days)		32	37.8	34.1
DHS onset to reflare, range (days)		11–82	12–71	11–82

*Note:* *Not a leprosy case but included in analysis as patient took MDT (dapsone included) for false diagnosis of leprosy and DHS occurred.

One male patient had been incorrectly diagnosed with leprosy (recognized by late histopathology results) and prescribed for MDT treatment. His data were included in this analysis as the objective of this study was to investigate the clinical features of DHS. Of the 24 patient files analyzed, 71% exhibited reflare symptoms after cessation of dapsone. The remaining 29% of the patients had a normal gradual decline in DHS symptoms. As mentioned above, the data for the DHS onset duration were expressed in two ways in this study. First, durations from the start of MDT to the day of onset of any “suspicious” clinical symptoms that were preludes to full‐blown DHS. Second, the duration from MDT start to final recognition of DHS and cessation of dapsone (in MDT). As patients or sometimes supervising clinicians were unable to recognize the hypersensitivity/allergy symptoms, patients usually continued to take dapsone even after the appearance of DHS symptoms. The average duration from MDT initiation to the appearance of any suspicious symptom was 30 days (range 2–130 days), and the average duration from the start of MDT to final drug cessation was 44 days (range 3–145 days). Thus, patients took an average of 15 days of dapsone, even after the appearance of DHS symptoms. For patients who developed reflare symptoms even after dapsone cessation, reflare was observed, on average, 34 days after DHS onset and, on average, 20 days after dapsone cessation. Data were collected from all patients until their final discharge.

**Figure 2:**
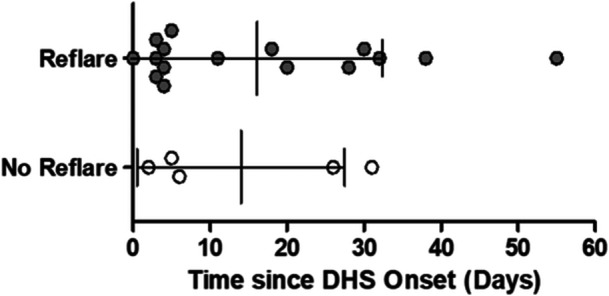
Cohort 1 time since DHS onset for reflare and no reflare DHS cases at which clinical data were available for comparison (16 vs. 5 shown here). Error bars indicate the mean with standard deviations. There was no difference in the time duration.

Fever (11/17, 65%) and skin eruptions (11/17, 65%) were the most common symptoms observed during reflare (Table 3). The next most common symptoms were itching (24%, 4/17) and jaundice (18%, 3/17). Burning micturition, tonsillitis, thrombophlebitis, herpes zoster, and angular stomatitis each occurred in 6% (1/17) of patients. When data were compared between patients who experienced reflare of symptoms (after dapsone cessation) and those who did not, it was found that patients who experienced reflare of symptoms reported longer DHS onset duration (average 35 days) than those who did not (average 16 days) (Mann–Whitney *U*, *p* = 0.049) (Table 4). This also led patients to take a longer duration of MDT before cessation (48 days vs. 36 days, *p* = 0.39). Comparisons were also made based on the first laboratory data available at the time of DHS onset (independent of the reflare occurrence and thus may not coincide with peak organ involvement). Not all patients had laboratory data, which hindered the effective determination of statistical significance. Days since DHS onset at which laboratory data were available are shown in Figure 2. No significant difference on time of any laboratory data available was present between those who had reflare and who did not (median days (range): 8 (0–55) vs. 6 (2–31), respectively, *p* > 0.05) (Figure 1). Despite this, it was found that the reflare group had a less favorable outcome than the non‐reflare group. Hemoglobin was lower (10.7 g/dL vs. 12.1 g/dL, *p* = 0.108), total bilirubin higher (5.6 mg/dL vs. 3.5 mg/dL, *p* = 0.701), direct bilirubin higher (4.1 mg/dL vs. 2.1 mg/dL, *p* = 0.645), OT or AST higher (140.9 U/L vs. 70.5 U/L, *p* = 0.762), PT or ALT higher (255.5 U/L vs. 71.0 U/L, *p* = 0.231) and alkaline phosphatase higher (418.0 U/L vs. 168.8 U/L, *p* = 0.212) in the reflare group compared to the non‐reflare group.

**Table 3:** Types of symptoms during reflare of DHS symptoms (Cohort 1).

Variables	Females						Males											Reflare symptoms frequency	
Age (years)	41	46	51	77	15	30	15	18	18	21	43	48	28	43	30	56	21		
Reflare symptoms	F1	F2	F3	F4	F5	F6	M1	M2	M3	M4	M5	M6	M7	M8	M9	M10	M11	*n*	%
Fever	1	1	1		1		1	1		1		1	1			1	1	11	64.7
Exfoliation/plaques	1			1		1	1		1	1	1	1	1		1		1	11	64.7
Burning micturation		1																1	5.9
Jaundice			1							1		1						3	17.6
Itching				1					1				1		1			4	23.5
Tonsillitis								1										1	5.9
Thrombophlebitis								1										1	5.9
Herpes zoster														1				1	5.9
Angular stomatitis															1			1	5.9

*Note:* M and F indicate male and female cases, respectively.

**Table 4:** Comparison of clinical and laboratory data between groups who reflared and those who did not.

	Reflare not seen	Reflare seen	Exact Wilcoxon rank sum test		
*N*	7	17	Data available*	*W*	*p*
Age, years, avg. (SD)	43.4 (22.3)	35.4 (17.3)	7 vs. 17	74	0.3736
Age range	20–67	15–77	7 vs. 17		
Male:female	1.33 (4/3)	1.83 (11/6)	7 vs. 17		
MDT to onset, days, avg. (SD)	15.7 (15.1)	34.8 (27.1)	6 vs. 17	23	**0.049**
MDT to drug cessation, days, avg. (SD)	36.1 (33.3)	47.8 (28.9)	7 vs. 17	45.5	0.391
Hemoglobin, g/dL, avg. (SD)	12.1 (1.3)	10.7 (2.2)	5 vs. 12	45.5	0.108
Total bilirubin, mg/dL, avg. (SD)	3.5 (3.0)	5.6 (7.2)	5 vs. 14	30.5	0.701
Direct bilirubin, mg/dL, avg. (SD)	2.1 (2.7)	4.1 (6.5)	5 vs. 14	25	0.645
OT/AST, U/L, avg. (SD)	70.5 (55.1)	140.9 (194.2)	4 vs. 12	23	0.761
PT/ALT, U/L, avg. (SD)	71.0 (36.6)	255.5 (290.6)	3 vs. 9	7.5	0.230
Alkaline phosphatase, U/L, avg. (SD)	168.8 (118.4)	418.0 (353.4)	4 vs. 11	13	0.212

*Note:* Lab tests analyzed here were the first available lab test results after DHS onset and the time of tests for various lab parameters could vary. Thus, the test values may not coincide with the peak severity during DHS episodes. Laboratory tests were not available for all patients, and the results shown are based on the data available. Bold value is statistically significant.

* Not all patients had lab data available, comparisons were done based on available data.

### 3.2 Molecular Epidemiology

#### 3.2.1 Specificity Analysis of HHV Primers

**Figure 3:**
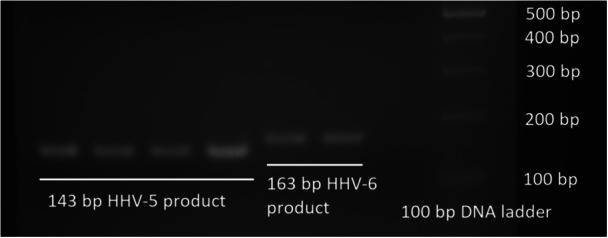
Gel electrophoresis of PCR product of few HHV‐5 and HHV‐6 positive (qPCR) samples. In silico analyses showed that HHV‐5 primers produced a 143 bp product and HHV‐6 primers produced a 163 bp product.

**Figure 4:**
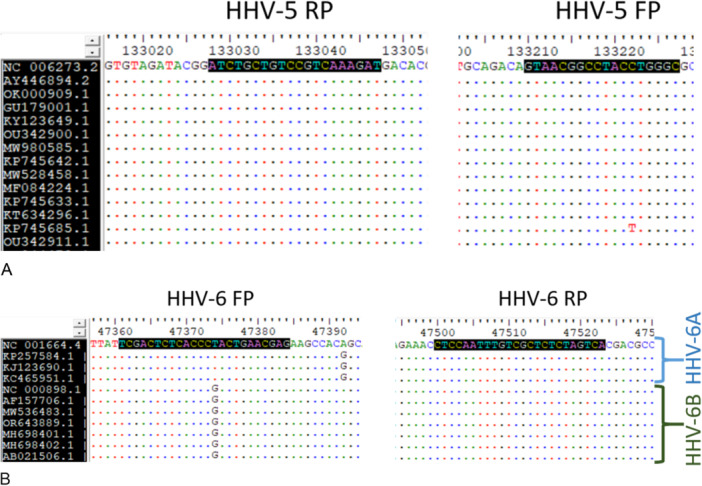
Position of primers in some HHV‐5 (A) and HHV‐6A and 6B (B) genomes (FP: forward primer, RP: reverse primer).

The HHV primers used in this study amplified the respective virus species. In silico sequence analyses showed that the HHV‐5 [33] and HHV‐6 [34] primers corresponded to the respective virus sequences in GenBank. Attempts were made to download HHV‐5 and HHV‐6 (HHV‐6A and HHV‐6B) virus sequences (Source: NCBI Virus) from viruses isolated from different regions of the world (Europe, USA, Asia, and Africa) and from different time periods (before 2000 AD, 2000s, 2010s, and the 2020s) (data not shown). Analyses showed that HHV‐5 primers amplified a 143 bp product and HHV‐6 (HHV‐6A and HHV‐6B) primers amplified a 163 bp product. HHV‐5 primers amplified the “DNA polymerase catalytic subunit” gene (PP386308.1), whereas HHV‐6 primers amplified the “large tegument protein deneddylase” gene (KY315558.2). Almost all HHV‐5 sequences were bound by the forward and reverse primers. For HHV‐6A, both forward and reverse primers bound perfectly, whereas the 14th nucleotide in the forward primer had a mismatch in HHV‐6B (FP should have been G instead of T for a perfect match) genomes (Figure 3). Representative matches of the primers are shown in the figures below (Figures 3A,B and 4).

#### 3.2.2 Detection of Herpesvirus DNA in DHS Patient Samples (Cohort 2)

**Figure 5:**
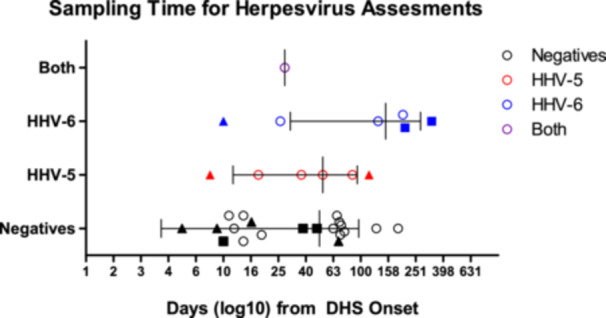
Cohort 2 distribution of time (in days) at which blood samples were collected for herpesvirus detection. Black, red, blue, and purple colors, respectively, indicate negative, HHV‐5/CMV positive, HHV‐6 positive, and both HHV‐5 and HHV‐6 positive. Solid triangles and squares, and open circles, respectively, denote reflare recorded, no reflare recorded, and no reflare data available. Error bars show the mean and standard deviation.

**Figure 6:**
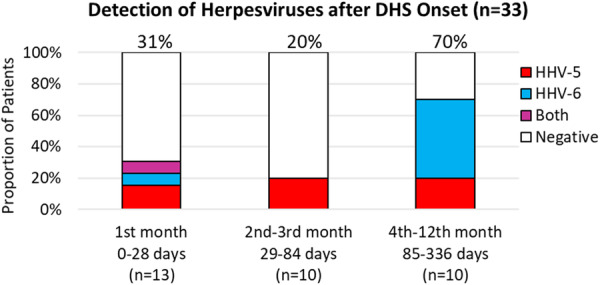
Proportions of herpesvirus (HHV‐5 and HHV‐6) positivity in samples collected at various time windows after DHS onset.

**Figure 7:**
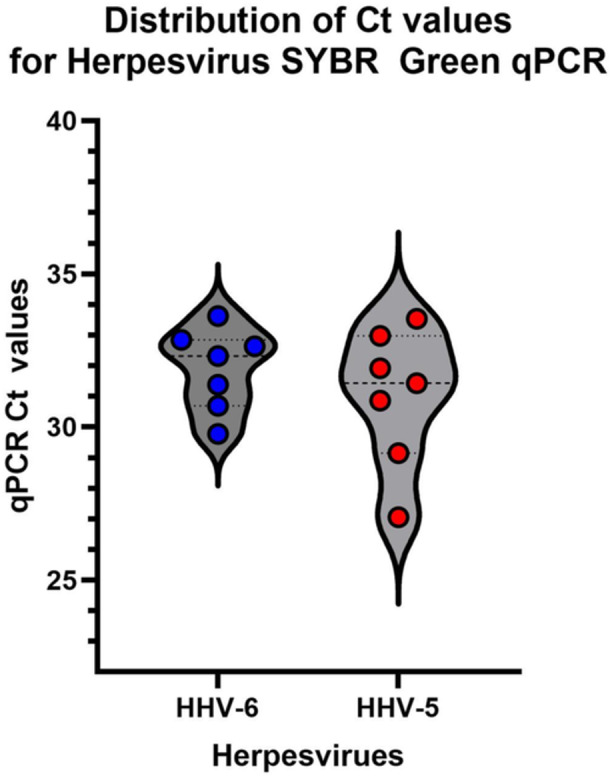
Distribution of cycle threshold (Ct) values for HHV‐5 and HHV‐6 SYBR Green qPCR.

SYBR‐green‐based qPCR was performed on DNA extracted from 33 patient blood samples collected in EDTA tubes. β‐actin was run in all samples to confirm satisfactory extraction (Ct < 25). Thirty‐three patient samples obtained 1 week to 1 year after DHS onset were used to investigate the presence of herpesviruses HHV‐5/CMV and HHV‐6 (Figure 5). The male‐to‐female ratio was 1.5 (20/13). The mean ages ± std. dev of all, male and female participants were 31 ± 16 years, 29 ± 12 years, and 34 ± 20 years, respectively. Samples were collected on average 75.6 days (median 62 days, range 5–328 days) after DHS onset. A total of 39.4% (13/33) of patients were positive for at least one herpesvirus (Figure 6). Of the samples collected within 2 weeks of onset, 22% (2/9) were positive for at least one of the two herpesviruses. Of the samples collected within 1 month of DHS onset, 31% (4/13) were herpesvirus‐positive. Similarly, 20% (2/10) of samples collected from the 2nd to 3rd month and 70% (7/10) of samples collected from the 3rd month to 1 year were positive for at least one of the two herpesviruses. The mean Ct values (cycle threshold) for HHV‐5 and HHV‐6 were 31.0 (range 27–33.5) and 31.9 (range 29.8–33.6), respectively (Figure 7). The presence of these two herpesviruses was also examined in 30 blood samples from new leprosy cases before the start of dapsone/MDT treatment. None of the samples (*n* = 30) were positive for either herpesviruses compared to 13/33 in Cohort 2 (Fisher's Exact test, *p* < 0.0001) (data not shown).

Furthermore, the medical charts for Cohort 2 were reviewed (see Table 5). Of the 33 patients with DHS, 36% (12/33) were admitted to Anandaban Hospital (study site) during their DHS episode, and thus, more detailed clinical and laboratory data were available (Group A). For the remaining 64% (21/33) who were admitted elsewhere during their DHS episode, the data were scant (Group B). In Group A, 58% (7/12) experienced a reflare episode. Of the reflare‐positive patients, 43% (3/7) were herpesvirus‐positive compared to 40% (2/5) of reflare‐negative patients (*p* > 0.05). Two of the three (67%) herpesvirus‐positive samples in the reflare group were HHV‐5 positive, compared to none (two HHV‐6 positives only) in the no reflare group. Overall, 42% (5/12) of patients in Group A and 38% (8/21) in Group B were herpesvirus‐positive.

**Table 5:** Herpesvirus positivity in Cohort 2.

				All cases (%)			Reflare positive cases (%)				Reflare negative cases (%)			
	Sampling time post onset: month (days)	No. of cases (% of 33)	No. of cases (%)	HHV+ (%)	HHV‐5+ (%)	HHV‐6+ (%)	Reflare positive cases (%)	Reflare positive and HHV+ (%)	Reflare HHV‐5+ (%)	Reflare HHV‐6+ (%)	Reflare negative cases (%)	Reflare negative & HHV+ (%)	Reflare negative HHV‐5+ (%)	Reflare negative HHV‐6+ (%)
Group A (12)	1 (1–28)	6/33 (18%)	6/12 (50%)	2/12 (17%)	1/12 (8%)	1/12 (8%)	5/12 (42%)	2/12 (17%)	1/12 (8%)	1/12 (8%)	1/12 (8%)	0/12	0/12	0/12
	2 (29–56)	2/33 (6%)	2/12 (17%)	0/12	0/12	0/12	0/12	0/12	0/12	0/12	2/12 (17%)	0/12	0/12	0/12
	3 (57–84)	1/33 (3%)	1/12 (8%)	0/12	0/12	0/12	1/12 (8%)	0/12	0/12	0/12	0/12	0/12	0/12	0/12
	5 (113–140)	1/33 (3%)	1/12 (8%)	1/12 (8%)	1/12 (8%)	0/12	1/12 (8%)	1/12 (8%)	1/12 (8%)	0/12	0/12	0/12	0/12	0/12
	8 (197–224)	1/33 (3%)	1/12 (8%)	1/12 (8%)	0/12	1/12 (8%)	0/12	0/12	0/12	0/12	1/12 (8%)	1/12 (8%)	0/12	1/12 (8%)
	12 (307–336)	1/33 (3%)	1/12 (8%)	1/12 (8%)	0/12	1/12 (8%)	0/12	0/12	0/12	0/12	1/12 (8%)	1/12 (8%)	0/12	1/12 (8%)
	Group A Total	12/33 (36%)	12/12 (100%)	5/12 (42%)	2/12 (17%)	3/12 (25%)	7/12 (58%)	3/12 (25%)	2/12 (17%)	1/12 (8%)	5/12 (42%)	2/12 (17%)	0/12	2/12 (17%)
Group B (21)	1 (1–28)	7/33 (21%)	7/21 (33%)	2/21 (10%)	2/21 (10%)	1/21 (5%)								
	2 (29–56)	1/33 (3%)	1/21 (5%)	1/21 (5%)	1/21 (5%)	0/21								
	3 (57–84)	6/33 (18%)	6/21 (29%)	1/21 (5%)	1/21 (5%)	0/21								
	4 (85–112)	2/33 (6%)	2/21 (10%)	2/21 (10%)	1/21 (5%)	1/21 (5%)								
	5 (113–140)	2/33 (6%)	2/21 (10%)	1/21 (5%)	0/21	1/21 (5%)								
	7 (169–196)	1/33 (3%)	1/21 (5%)	0/21	0/21	0/21								
	8 (197–224)	1/33 (3%)	1/21 (5%)	1/21 (5%)	0/21	1/21 (5%)								
	9 (225–252)	1/33 (3%)	1/21 (5%)	0/21	0/21	0/21								
	Group B Total	21/33 (64%)	21/21 (100%)	8/21 (38%)	5/21 (24%)	4/21 (19%)								
	Total Cohort (A + B)	33/33 (100%)	33/33 (100%)	13/33 (39%)	7/33 (21%)	7/33 (21%)								

*Note:* Group A patients were admitted to Anandaban at DHS onset and thus had chart details to determine whether a reflare occurred. Group B patients were admitted elsewhere at DHS onset and later referred to Anandaban without sufficient chart details to determine if a reflare had occurred.

## 4 Discussion

Drugs used to treat human diseases are usually small organic chemical molecules, a few of which have special chemical functional groups that may induce hypersensitivity reactions [36]. Many drug‐induced adverse reactions may be mild, such as common drug rashes and urticaria [37]. Severe cutaneous adverse reactions (SCARs) include Acute Generalized Exanthematous Pustulosis (AGEP), Drug‐Induced Hypersensitivity Syndrome/Drug Reaction with Eosinophilia and Systemic Syndromes (DiHS/DRESS), and Stevens–Johnson Syndrome (SJS)/Toxic Epidermal Necrolysis (TEN). These SCARs have severe symptoms, particularly manifested in the skin, mucus membranes, and internal organs. HHV‐6 reactivation has been repeatedly reported in association with DiHS/DRESS (Table 6). However, the association between DHS and herpesviruses has rarely been reported. DHS was first described in the African population in Nigeria (2.5%–4.5% [28, 38]).

**Table 6:** Clinical features of severe cutaneous adverse reactions (SCARs).

Severe cutaneous adverse reactions (SCARs)	Time to onset after drug intake	Mortality	HHV‐6 reactivation
Acute Generalized Exanthematous Pustulosis (AGEP)	< 2 days	5%	Not seen
Drug‐Induced Hypersensitivity Syndrome/Drug Reaction with Eosinophilia and Systemic Syndromes (DiHS/DRESS)	2–6 weeks	10%	Common
Steven–Johnson Syndrome (SJS)/Toxic Epidermal Necrolysis (TEN)	4–28 days	25%	Uncommon

Ishida et al. [23] studied the DNA viral loads for EBV, CMV, and HHV‐6 in patients of DiHS/DRESS, SJS, TEN, and SJS/TEN. HHV‐6 viral loads reached peaks at days ranging from 11th to 100th for DiHS/DRESS and HHV‐5 loads peaked at 31–100 days. HHV‐6 reactivation is used as a defining criterion by the Japanese Consensus Group but not by the RegiSCAR group [20]. Despite disagreement, both groups agree on the role of HHV‐6 reactivation in the association of increased severity of DRESS symptoms coinciding with “reflare” symptoms [17, 20, 39]. In fact, it has been noted that HHV‐6 reactivation during DiHS/DRESS could be the major contributor of severity [40].

This study was designed as an exploratory investigation to examine whether clinical reflare patterns and molecular detection of herpesviruses could be observed in patients with DHS, especially in a real‐world, resource‐limited setting. Given the retrospective nature of the chart review (Cohort 1), opportunistic sampling, absence of paired longitudinal samples (Cohort 2), and the use of independent cohorts for clinical and molecular analyses, the study does not establish causality, but rather generates hypotheses regarding the potential role of herpesvirus reactivation in DHS. This study provides preliminary clinical and molecular evidence for the presence of clinical reflare/reactivation of herpesviruses during the etiology of DHS (although in an independent group of patients). In Cohort 1, 71% of our patients showed reflare of symptoms, on average, 34 days after DHS onset and 20 days after the cessation of MDT. In Cohort 2, 39% of the DHS patient samples were positive for at least one of the herpesviruses. Availability of clinical data for patients in Cohort 2 was limited. Only 12/33 (36%) were initially admitted at Anandaban Hospital upon DHS diagnosis; and, of these, 7/12 (58%) experienced reflare episodes (Figure 5). The remainder of Cohort 2 arrived at Anandaban after DHS diagnosis elsewhere with limited to no medical chart data. Thus, the prevalence of reflare and relevant timing since DHS onset or diagnosis could not be determined. While herein, 71% (17/24) of Cohort 1 demonstrated reflare, 58% (7/12) in Cohort 2 developed reflare, of which 2 HHV‐5 and 1 HHV‐6 positivity could be detected. Therefore, within the limitations of this study, 43% (3/7) demonstrated HHV positivity at their different individual times of sampling; and reflare was shown to occur in DHS cases with HHV‐5 reactivation. Additionally, Shiohara et al. [20] mentioned reactivation of HHV‐6 may take on average 2–3 weeks after onset, very similar to the reflare time of average 20 days since dapsone cessation seen in our study. Our study found a concentration of HHV‐5 positivity in earlier days (within 100 days since onset) compared to HHV‐6 positivity which was spread across 1 year duration. Earlier study mentioned somewhat earlier presentation of HHV‐6 compared to HHV‐5 [23, 41]. Our study was limited by opportunistic one‐time sampling and thus positivity may also represent remnant viruses (high Ct values seen) after initial reactivation. Despite that, we also observed both HHV‐5 and HHV‐6 positivities in the same time window, also reported in literature [20, 23].

Symptom reflare may be due to herpesvirus reactivation [16, 20, 42, 43]. Another support to the evidence of reflare comes from the fact that liver severity was higher in the reflare group than in the non‐reflare group, although the differences were not statistically significant. Higher severity is a known variable for herpesvirus reactivation, especially for herpesvirus‐5/CMV [26]. Our retrospective analysis found worse liver lab outcomes in the reflare group than in the non‐reflare group, which is similar to a previous study [43]. In Cohort 1, there was a significant difference in the duration of DHS onset between the reflare and non‐reflare groups. The reflare and non‐reflare groups were small in size, and the statistically significant difference in DHS onset duration was an unexpected finding. The degree of detail included in the chart notes varied among clinicians and over time. To address this possible limitation, all minor details—specifically, the days of DHS onset and drug cessation—were carefully recorded during the chart review process to provide objective inferences. Longer DHS onset durations may have provided more stimulation for the drug‐induced proliferation of T cells or immunological stressors, which are known reactivators for HHVs [44, 45].

## 5 Limitations and Bias

As most leprosy patients registered at Anandaban Hospital come from various places in Nepal and return home after leprosy diagnosis for continuation of MDT medication at their local health facilities, many patients who developed DHS might have been treated in healthcare facilities near their homes. Therefore, this study might not provide an accurate estimation of the prevalence of reflare symptoms in patients with DHS, but only a trend. In Cohort 1, there were significant differences in the onset durations of DHS in reflare and non‐reflare groups. However, as fewer lab tests were taken in previous years (early 2000s), there was insufficient sample size to detect statistical differences for comparison of lab values. Similarly, in Cohort 2, blood samples were taken at widely different, opportunistic times (convenience sampling). Thus, HHV‐5 and HHV‐6 results are not indicative of when HHV reactivation occurred in relation to the DHS episode or reflare, but rather indicate which cases were positive by that specific time for each individual. Therefore, the herpesvirus prevalence data reported in this study may not represent the timing or prevalence of herpesvirus reactivation during a DHS episode. In future, more systematic sampling could potentially determine differences in time between HHV reactivation relative to clinical reflare. Only HHV‐5 and 6 were examined because of their roles in the severity [40] and etiology [18] of DHS. Future studies are planned with a more systematic research design. Examination of all possible HHVs (HHV‐1 through HHV‐7) regularly (e.g., weekly or biweekly) for a definite interval of time could provide a more comprehensive picture of HHV reactivation in DHS. Additionally, the impact of antivirals on drug allergies could be studied through clinical trials.

Another major limitation of this study was the unavailability of paired samples for HHV detection at the time of reflare. This could have provided vital evidence regarding the timing of reflare and reactivation. However, our blood samples in Cohort 2 were collected as part of another study not strictly focused on HHV detection. Most of the blood samples were collected after 2017, whereas our chart review (Cohort 1) consisted of cases since 2000–2018. This limitation can lead to serious questions regarding whether reflare symptoms represent HHV reactivation. However, as discussed above, there is a high chance that reflare or worsening of severity coincides with HHV reactivation [20, 42, 43]. Another possibility of reflares in the DHS patients could be due to occurrence of leprosy reactions. However, the chances of these reflares actually being leprosy reactions are minimal because being a leprosy referral center with experienced dermatologists, the clinicians are highly vigilant on whether the reflare symptoms match the well‐known criteria of leprosy reactions [31] or with sequelae of DHS. Additionally, reflares are also reported in allergies due to other drugs where leprosy reactions‐like phenomena are not present. We did not analyze the differences in the severity of patients with or without HHV, as HHV negativity could have occurred due to limitations of opportunistic timing of blood collection. However, upon review of Cohort 2 patient files, only 36% (12/33) of the patients were admitted to our hospital; thus, an appropriate comparison was not possible. Similarly, our study was also limited to the detection of HHV‐5/CMV and HHV‐6 only, while the literature has also reported the reactivation of EBV, herpes zoster, and other herpesviruses. As this was a preliminary study, we investigated two major herpesviruses. HHV‐6 has been widely reported and is a defining criterion for DRESS/DiHS by the Japanese consensus group. Similarly, HHV‐5/CMV is a major HHV associated with DRESS/DiHS severity. In addition, the forward primer of HHV‐6 did not perfectly bind HHV‐6B DNA targets due to a single mismatch in the middle of the primer. Considering the tolerability of Taq polymerase, especially when mismatch occurs farther from the 3′ sequence of the primers [46], we considered that the primers successively amplified the HHV‐6B targets.

An additional key limitation of this exploratory study is inherent with the methodology used. DNA‐based PCR is not able to distinguish between active or latent infection, nor can it differentiate chromosomally integrated herpesviruses from replicating viruses [47]. Given the resource constraints and high sensitivity and specificity of the nucleic acid amplification test, PCR was employed to detect the herpesviruses in this study. Notably, herpesviruses were not detected in all DHS patients (only ~40% positive) and were also absent in the newly diagnosed leprosy cases. This provides validity to the method used as absence of herpesvirus DNA in new leprosy cases suggests that herpesvirus DNA detection in DHS cases is unlikely to reflect background carriage alone. This study provided preliminary evidence, at the DNA level, suggesting differences in herpesvirus detection patterns in DHS cases. Although herpesvirus reactivation during drug allergies is a common phenomenon recognized by the scientific community, very little data are available on DHS cases [26]. Chen et al. [24] examined six of their nine enrolled Taiwanese DHS patients but were unable to detect any HHV‐6/EBV positives. The study herein enrolled 33 patients with DHS and found HHV‐5/6 in 39% of them.

## 6 Conclusion

In alignment with the known phenomenon of HHV reactivation during drug hypersensitivity/allergy, our data provide preliminary evidence for HHV‐5 and HHV‐6 reactivation in Nepalese DHS cases. We reviewed the medical charts of patients with DHS admitted to our hospital at the time of DHS onset. Similarly, we used qPCR to assess the presence of HHV‐5 and 6 after the onset of DHS. The data presented here demonstrate the detectable presence of HHV‐5 or 6 in a subset of DHS patient samples. A major limitation of our study was the limited availability of matched clinical reflare data with the qPCR results. The two sets (Cohorts 1 and 2) of analyses were carried out in entirely different groups of patients owing to the paucity of samples. Despite that, patients in both cohorts were similar with respect to demographic and clinical features. DHS is a rare phenomenon (2%–3%), and patients arrive at our hospital at different points in the natural history of the disease/syndrome. It is difficult to confirm that the reflare of symptoms occurred due to reactivation of the herpesvirus, although such a connection has been reported. More systematic studies can provide more detailed answers on how and which herpesviruses play a role in the natural history or severity of DHS.

## Author Contributions

**Divya RSJB Rana:** conceptualization, data curation, formal analysis, investigation, methodology, project administration, validation, visualization, writing – original draft, writing – review and editing. **Jivan Shakya:** data curation, formal analysis, investigation, methodology, project administration, resources, validation, writing – review and editing. **Suwash Baral:** data curation, investigation, writing – review and editing. **Reejana Shrestha:** data curation, investigation, writing – review and editing. **Kishor Koju:** data curation, investigation, methodology. **Jarina Joshi:** conceptualization, investigation, supervision, resources, writing – review and editing. **Deanna A. Hagge:** conceptualization, formal analysis, investigation, methodology, resources, supervision,validation, visualization, writing ‐ original draft, writing – review and editing. **Mahesh Shah:** conceptualization, data curation, formal analysis, investigation, project administration, resources, validation, supervision, writing – review and editing.

## Ethics Statement

The study was conducted according to the prevailing principles of Good Clinical Practice and the Declaration of Helsinki. Ethics approval was obtained from Nepal Health Research Council, Kathmandu, Nepal.

## Consent

Formal consent (verbal and written) was obtained prior to sample collection.

## Conflicts of Interest

The authors declare no conflicts of interest.

## Data Availability

The data that support the findings of this study are available from the corresponding author upon reasonable request.
